# Psychotropic Polypharmacy in Adults 55 Years or Older: A Risk for Impaired Global Cognition, Executive Function, and Mobility

**DOI:** 10.3389/fphar.2019.01659

**Published:** 2020-01-30

**Authors:** Gilles Loggia, Elpidio Attoh-Mensah, Kristell Pothier, Rémy Morello, Pascale Lescure, Marie-Laure Bocca, Christian Marcelli, Chantal Chavoix

**Affiliations:** ^1^Normandie Univ, UNICAEN, INSERM, COMETE, Caen, France; ^2^Department of Geriatrics, Normandie Univ, UNICAEN, CHU de Caen Normandie, Caen, France; ^3^Department of Statistics and Clinical Research, Normandie Univ, UNICAEN, CHU de Caen Normandie, Caen, France; ^4^Department of Rheumatology, Normandie Univ, UNICAEN, CHU de Caen Normandie, Caen, France

**Keywords:** polypharmacy, psychotropic drugs, cognition, executive function, gait

## Abstract

**Objectives:**

With their broad spectrum of action, psychotropic drugs are among the most common medications prescribed to the elderly. Consequently, the number of older adults taking multiple psychotropic drugs has more than doubled over the last decade. To improve knowledge about the deleterious effects of psychotropic polypharmacy, we investigated whether there is a threshold number of psychotropic molecules that could lead to impairment of global cognition, executive function, or mobility. Furthermore, relationships between the number of psychotropic molecules and cognitive and mobility impairment were examined.

**Design:**

Cross-sectional study

**Setting:**

University Hospital of Caen (France) and advertisements in medical offices

**Participants:**

Community-dwelling older adults 55 years and older (n = 177; 69.8 ± 9.3 years; 81% women)

**Measurements:**

Number of psychotropic molecules taken daily, global cognition assessed with the Mini Mental State Examination (MMSE) and the Montreal Cognitive Assessment (MoCA), processing speed with the Trail Making Test (TMT) A, executive function with the TMT B and TMT B-A, and mobility with the Time Up and Go (TUG). The threshold numbers of psychotropic molecules were determined by ROC curves analysis. Based on these threshold values, multinomial logistic regression adjusting for covariates was then performed.

**Results:**

Logistic regressions showed that the threshold of two daily psychotropic molecules, identified by the ROC curves analysis, increases the risk of impaired executive function (*p* = .05 and.005 for the TMT B and TMT B-A, respectively), global cognition (*p* = .006 and.001 for the MMSE and MoCA, respectively), and mobility (*p* = .005 for the TUG), independent of confounding factors, including comorbidities. Furthermore, psychotropic polypharmacy would affect mobility through executive functions.

**Conclusion:**

Impairment of global cognition, executive function, and mobility when as few as two psychotropic molecules are consumed in relatively healthy young older adults should alert physicians when prescribing combinations of psychotropic medications.

## Introduction

Polypharmacy, usually defined as the concomitant daily use of five or more medications (Gnjidic et al., 2012), *is i*ncreasingly common. It is, however, associated with serious adverse events such as falls, frailty, disability, and mortality in older adults (Lai and Liao, 2013; Maher et al., 2014; Moulis et al., 2015). Polypharmacy increases the risk of falls by as much as five times(Montero-Odasso et al., 2005; Woolcott et al., 2009; de Groot et al., 2013; Montero-Odasso et al., 2019), in part through gait disturbances (Montero-Odasso et al., 2019). Polypharmacy also impairs cognition (Langeard et al., 2016; Rawle et al., 2018). Yet, association between low cognitive abilities and increased risk of falling is well-known (Herman et al., 2010; Montero-Odasso et al., 2012; Li et al., 2018), which is consistent with the high interference of cognitive demands with postural control, widely demonstrated in both young and older adults (Lundin-Olsson et al., 1997), and even more so in old-old adults (Pothier et al., 2015). Among the cognitive abilities, executive function would be preferentially involved in falls (Herman et al., 2010; Segev-Jacubovski et al., 2011; Hsu et al., 2012). We previously showed that community-dwelling older adults 55 years and over who took five or more medicinal molecules per day were at high risk for both impaired global cognition and mobility (Langeard et al., 2016); however, executive function and specific involvement of the different pharmacological classes were not investigated.

Psychotropic drugs (i.e. antidepressants, mood stabilizers, anxiolytics, antipsychotics, and various analgesics) are among the most common medications prescribed to the elderly (Bareis et al., 2018). In 2016, 25% of seniors 65 years and older and living at home, as well as 74% of those in institutions, received boxes of psychotropic drugs (INSEE References, 2018). This can be explained by their broad spectrum of action (WHO Collaborating Centre for Drug Statistics Methodology, 2019). For instance, antidepressants are prescribed for a wide array of illnesses besides depression (e.g. chronic pain, anxiety, smoking cessation), and anxiolytics mainly to treat anxiety but also for sleep disorders that are continually rising (Hartikainen et al., 2005).

As a consequence, the number of older adults taking multiple psychotropic drugs has more than doubled over the last decade. Thus, 12% of psychotropic users take at least two psychotropic drugs (Hartikainen et al., 2003), and one in four elderly persons uses analgesics and psycholeptics or antidepressants concomitantly (Hartikainen et al., 2005). Because psychotropic drugs put users at risk for falls (Bloch et al., 2011; Curkovic et al., 2016) and affect cognition (Brooks and Hoblyn, 2007; Vetrano et al., 2018), they may be significantly involved in the adverse effects of polypharmacy. This would be consistent with the associations between the use of multiple central nervous system medications and 1.5- to 2.4-fold increased fall risk (Weiner et al., 1998), and the increased risk of fall injuries, hospitalization, and death in a dose-response manner with the use of four psychotropics (Johnell et al., 2016). The adverse effects of psychotropic polypharmacy, i.e., two or more psychotropic drugs, on cognition and mobility, and the possible links between both outcomes when taking psychotropic polypharmacy are thus important issues to address.

To this aim, we conducted an in-depth investigation on the effects of psychotropic drugs on global cognition, processing speed, executive function, and mobility performance. The main objective was to determine whether there is a threshold number of psychotropic molecules that could lead to cognitive or mobility impairment using a statistical method for risk prediction. Furthermore, relationships between the number of psychotropic molecules and cognitive and mobility impairment were examined.

## Methods

### Patients

We included 177 community-dwelling adults 55 years and over in the study as part of a hospital clinical research program whose main objective was to investigate the role of osteoporosis and cognitive impairment in fall-related fractures in seniors. The age of 55 years was chosen as that from which fractures resulting from osteoporosis become increasingly common in women (Compston et al., 2019), which is also an age when some cognitive abilities have often begun to decline. Participants were recruited through the orthopedic and emergency departments at the university hospital of Caen (France) and advertisements in medical offices, from May 2011 to May 2017, following a low-energy fall, with or without fracture, in the year prior to the study. Exclusion criteria were as follows: inability to walk alone for 15 meters without help, pathology affecting balance, neurodegenerative or related pathology, drinking more than 21 units of alcohol per week (14 for women), and impaired vision (acuity <6/10). The Lower Normandy Ethics Committee approved the present study (no. 2011A00556-35; clinical trial registration number: NCT02292316), and each participant provided written informed consent. The present study focused on the cross-sectional outcomes obtained from the prospective cohort study.

### Outcome Measures

During the medical examination, drug treatment was meticulously noted from prescriptions and confirmed by medical history. The following data were also collected: socio-demographic data, comorbidities (the 12 items from the Kaplan-Feinstein scale (Kaplan and Feinstein, 1974) such as hypertension, cardiac, central nervous system, or locomotion), number of risk factors for falling (e.g., hypotension, rheumatological disorders, muscular weakness, and abnormal proprioceptive sensitivity in the lower limbs), body mass index (BMI), and muscular strength as measured by a handgrip dynamometer. Trained experimenters, blind to the participants’ medical treatment, performed cognitive and mobility evaluations.

Global cognitive performance was assessed with the Mini Mental State Examination (MMSE) and the Montreal Cognitive Assessment (MoCA). The cut-off score for impairment was set at 24/30 for the MMSE (Folstein et al., 1975) and 26/30 for the MoCA (Nasreddine et al., 2005). Processing speed was evaluated by the Trail Making Test (TMT) A, and executive function by the TMT-B. The TMT-A consists of connecting numbers, randomly displayed on an A4 sheet of paper, in ascending order, as quickly as possible. The TMT-B requires the subject to alternate between numbers (in ascending order) and letters (in alphabetical order) which involves mental flexibility. The completion time for each part of the TMT was recorded (further called TMT A and TMT B), and the difference score (TMT B-A) was used as a relatively pure indicator of executive control abilities (Sanchez-Cubillo et al., 2009). The presence of a deficit for each three scores was identified from the normative data stratified by age and education (Senior et al., 2018).

Mobility performance was assessed with the Time Up and Go (TUG) that requires standing up from an armchair, walking 3 meters, turning, walking back, and sitting down, all at a comfortable pace; two trials were attempted, and the shortest time to complete the task was recorded. Impaired TUG was based on the normative reference values corrected for age (Bohannon, 2006).

### Identification of the Psychotropic Molecules

The total number of molecules (e.g., two molecules in case of the combination of two molecules in a single tablet) that were taken per day by each participant was first determined and used to calculate medical exposure. Medications were then classified based on the Anatomical Therapeutic Chemical (ATC) classification, developed by WHO, to identify the pharmacological molecules with psychotropic properties, defined here as having an effect on the nervous system, i.e., anesthetics (N01), analgesics (N02), anti-epileptics (N03), anti-Parkinson drugs (N04), psycholeptics (N05), and psychoanaleptics (N06) (WHO Collaborating Centre for Drug Statistics Methodology, 2019). Analgesics that belong to the N02B subclass (e.g. acetylsalicylic acid, paracetamol) were excluded because they are not considered as psychotropic molecules.

### Statistical Analysis

Comparisons between characteristics of users and non-users of psychotropic molecules were performed using a two-sided Student’s *t-*test or Mann Whitney U test, and a Chi-square test or Fisher’s exact test when appropriate. The normality of the data distribution was evaluated by the Kolmogorov-Smirnov test, and, if necessary, were log-transformed before analysis to meet requirements for normal distributions. Then, we used adjusted Pearson correlations to investigate the relationship between the number of daily psychotropic drugs taken and the variables studied. Adjustment variables were age, education, and comorbidities for the correlations with the cognitive scores (and age only for the MoCA scores already corrected for education), and age, handgrip strength, risks for falls, comorbidities, and BMI for correlation with TUG scores. The comorbidity items used as covariates were those that significantly differed between groups.

The receiver operating characteristic (ROC) curve was used to determine whether there is threshold numbers of psychotropic molecules that could lead to an impaired cognitive or mobility score (Delacour et al., 2005). The area under the ROC curve (AUC) provides a measure of accuracy of the prediction (Faraggi and Reiser, 2002).

Finally, based on the threshold values thus determined, we performed univariate multinomial logistic regression analyses on the backward selection method to find specific links between the number of psychotropic molecules and cognitive and mobility scores (impaired *vs* normal). The adjustment variables were those not included for the cut-off calculation (see above in “Outcome measures”). Only the adjusted variables with a regression *p*-value <15% were further used in the multivariate model. All analyses were performed with ﻿IBM SPSS (version 24.0; SPSS Inc., Chicago, IL, USA).

## Results

As shown in **Table 1**, mean age of the participants was 69.8 years ± 9.32. Participants were mostly women (81%), with high education (almost 12 years of schooling), and mean global cognitive and mobility scores (MMSE and MoCA, and TUG scores, respectively) within the range of normal values. Forty-one percent of the participants were taking psychotropic molecules, and 23% of these psychotropic users took a single psychotropic drug. Among psychotropic drugs, antidepressants was the class represented most frequently, closely followed by analgesics and hypnotics. There was no significant difference between men and women except for education (p = .01) and comorbidities (p = .049). The list of the psychotropic drugs and their frequency in the study population can be found in **Table 2**.

**Table 1:** Characteristics of the study population (n = 177).

	General population n = 177	Women n = 144	Men n = 33	*p*-value^#^
Age	69.79 ± 9.32	69.8 ± 9.49	69.76 ± 8.19	NS^a^
Education (years)	11.61 ± 4.00	11.15 ± 3.55	13.64 ± 5.17	.01^a^
Number of prescribed molecules	5.09 ± 3.98	5.10 ± 3.99	5.06 ± 4.04	NS^a^
Falls with injury, number (%)	112 (63)	104 (72)	8 (24)	.012^b^
Psychotropic molecules taken				
Psychotropic molecules, number (%)				
0 psychotropic molecule 1 psychotropic molecule 2 psychotropic molecules 3 psychotropic molecules 4 psychotropic molecules	101 (59) 43 (23) 18 (10) 14 (7) 1 (1)	81 (56) 33 (23) 16 (11) 13 (9) 1 (1)	20 (61) 10 (30) 2 (6) 1 (3) 0 (0)	NS^b^ NS^b^ NS^b^ NS^b^ NS^b^
Psychotropic molecules in ATC class, number (%)				
Antidepressants Analgesics Hypnotics Anxiolytics Antiepileptics Anti-dementia molecules Antipsychotics	33 (30) 23 (21) 20 (18) 17 (16) 11 (10) 4 (4) 1 (1)	26 (28) 20 (22) 18 (20) 16 (17) 9 (10) 2 (2) 1 (1)	7 (40) 3 (18) 2 (12) 1 (6) 2 (12) 2 (12) 0 (0)	NS^b^ NS^b^ NS^b^ NS^b^ NS^b^ NS^b^ NS^b^
MMSE score MoCA score	27.71 ± 2.45 26.41 ± 4.14	27.63 ± 2.54 26.41 ± 3.62	28.03 ± 2.17 27.27 ± 3.64	NS^a^ NS^a^
TUG score (sec)	9.41 ± 3.13	9.52 ± 3.35	8.97 ± 1.95	NS^a^
Impaired scores, number (%)				
MMSE	14 (7.9)	12 (8.3)	2 (6.1)	NS^a^
MoCA	51 (28.8)	45 (31.3)	6 (18.2)	NS^a^
TMT A, completion time	31 (17.5)	25 (17.4)	6 (18.2)	NS^a^
TMT B, completion time	27 (15.3)	22 (15.3)	5 (15.2)	NS^a^
TMT B-A, completion time	19 (10.7)	15 (10.4)	4 (12.1)	NS^a^
TUG	56 (31.6)	44 (30.6)	12 (36.4)	NS^a^

Unless indicated, values are mean ± SD; ^a^Mann-Whitney U test, ^b^Chi square test, NS, non-significant; SD, standard deviation; ^#^: Gender comparison

**Table 2:** List of psychotropic drugs and their frequency in the study population, n = 109.

Drugs and ATC code	number (%)
** N06A Antidepressants**	**33 (30.3)**
N06AB10 Escitalopram N06AB03 Fluoxetine N06AX16 Venlafaxine N06AX03 Mianserin N06AB05 Paroxetine N06AA04 Clomipramine N06AB04 Citalopram N06AX21 Duloxetine N06AA09 Amitriptyline N06AB06 Sertraline	9 (8.3) 5 (4.6) 5 (4.6) 3 (2.8) 3 (2.8) 2 (1.8) 2 (1.8) 2 (1.8) 1 (0.9) 1 (0.9)
N02A **Analgesics**	**23 (21.1)**
N02AX02 Tramadol N02AJ06 Codeine N02AA01 Morphine N02AB03 Fentanyl	14 (12.9) 6 (5.5) 2 (1.8) 1 (0.9)
N05C **Hypnotics**	**20 (18.3)**
N05CF02 Zolpidem N05CF01 Zopiclone N05CM09 Valerianae radix N05CD11 Loprazolam	11 (10.1) 7 (6.4) 1 (0.9) 1 (0.9)
N05B **Anxiolytics**	**17 (15.6)**
N05BA08 Bromazepam N05BA12 Alprazolam N05BA06 Lorazepam N05BA05 Potassium Clorazepate N05BB01 Hydroxyzine	8 (7.3) 4 (3.7) 3 (2.8) 1 (0.8) 1 (0.8)
N03A **Antiepileptics**	**11 (10.1)**
N03AX16 Pregabalin N03AE01 Clonazepam N03AG02 Valpromide N03AF01 Carbamazepine N03AX09 Lamotrigine	5 (4.6) 2 (1.8) 2 (1.8) 1 (0.9) 1 (0.9)
N06D **Anti-dementia molecules**	**4 (3.7)**
N06DX01 Memantine N06DA02 Donepezil N06DA03 Rivastigmine	2 (1.8) 1 (0.9) 1 (0.9)
N05A **Antipsychotics**	**1 (0.9)**
N05AL01 Sulpiride	1 (0.9)

**Table 3** shows that users of psychotropic drugs were significantly older (p < .001), took more daily medications (p < .001), had a higher number of comorbidities (*p* < .01), and lower muscular strength (*p < *.05) than non-users of psychotropic drugs. Cognitive and mobility scores were significantly and consistently poorer in users than in non-users of psychotropic drugs.

**Table 3:** Comparisons between the characteristics of users and non-users of psychotropic molecules.

	Non-users of psychotropic molecules n = 115	Users of psychotropic molecules n = 62	*p*-value
Age	67.77 ± 9.13	72.49 ± 8.74	<.001^b^
Education (years)	12.00 ± 4.07	11.09 ± 3.89	NS^b^
Falls with injury, number (%)	61 (60.40%)	51 (67.11%)	NS^c^
Risk factors of falls	0.80 ± 0.75	1.01 ± 1.11	NS^b^
Number of prescribed molecules	3.37 ± 3.02	7.39 ± 3.97	<.001^b^
Comorbidities	1.50 ± 1.28	2.09 ± 1.57	<.009^b^
Handgrip strength (kg)	23.13 ± 8.59	20.79 ± 8.13	.023^b^
BMI	27.20 ± 4.96	27.75 ± 5.09	NS^a^
MMSE score	28.29 ± 1.86	26.95 ± 2.96	.002^b^
MoCA score	27.48 ± 2.77	25 ± 5.14	<.001^b^
TMT A, completion time (sec)	36.24 ± 14.74	43.43 ± 22.87	.006 ^b^
TMT B, completion time (sec)	84.91 ± 38.78	108.73 ± 57.04	.001^b^
TMT B-A (sec)	49.29 ± 31.82	69.58 ± 49.54	.001^b^
TUG score (sec)	8.70 ± 1.75	10.36 ± 4.18	.002^b^

Unless indicated, values are mean ± SD; ^a^Student’s t-test, ^b^Mann-Whitney U test, ^c^Chi square test, NS (non-significant), SD (standard deviation).

In addition, the more the participants used psychotropic drugs, the lower their scores on the different cognitive and mobility tests. The correlations remained significant after controlling for potential covariates (MMSE: *r* = -.181, *p < *.05; MoCA: *r* = -.179, *p < *.05; time to completion on TMT B: *r = *.178, *p < *.05; TMT B-A: *r = *.188, *p < *.02; and TUG: *r = *.191, *p < *.02), except for the TMT A (r = .097; p = .20).

**Figure 1:**
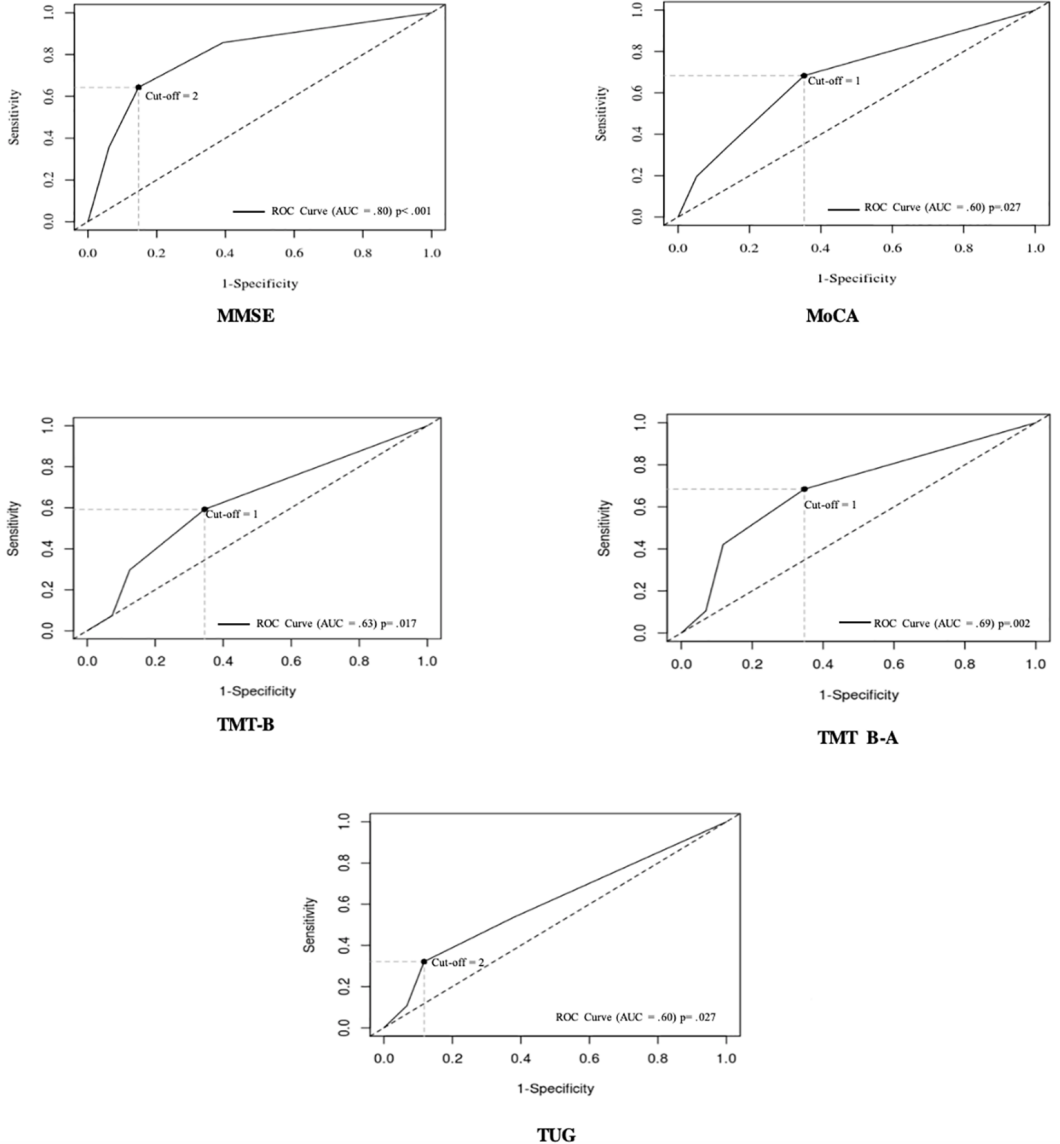
Receiver-operating characteristic (ROC) curves for the number of psychotropic molecules that predict impaired MMSE, MoCA, TMT B, TMT B-A, and TUG scores. Each point on the ROC curve indicates a specific cut-off, with each cut-off having its own sensitivity and specificity. The optimal cut-off is defined as the value, here that of the number or psychotropic molecules, that provides the best combination of sensitivity and specificity. This optimal cut-off can be identified as the intersection of the ROC curve with the upper left to lower right diagonal line. The area under the curve (AUC) is equal to 1 for perfect discrimination and 0.5 for an uninformative cut-off point.

Results from the ROC curves (**Figure 1**) revealed a threshold of one molecule for impaired MoCA, TMT B, and TMT B-A, with significant AUC (*p < *.05,.02, and.005, respectively), and two molecules for impaired MMSE and TUG, also with a significant AUC (*p* < .01 and.05, respectively).

We performed univariate multinomial logistic regression analysis based on these threshold values (0 *vs* ≥1 psychotropic molecules, and ≤1 *vs* ≥2 psychotropic molecules) to compare the risk of impaired cognition and mobility between participants, depending on their consumption of psychotropic molecules. It should be pointed out that impaired performance in participants was found in 31% for the TUG, 29% for the MoCA, 15% for the TMT A, 15% for the TMT, 11% for the TMT B-A, and 8% for the MMSE (**Table 1**). **Table 4** summarizes the results of the final models (Model 3) after taking at least one psychotropic and more than two psychotropic molecules. The analyzes showed that participants taking two or more psychotropic molecules had a significant increased risk for impaired MMSE (Model 3.1), MoCA (Model 3.2), TMT B (Model 3.4), TMT B-A (Model 3.5), and impaired TUG (Model 3.6), independent of confounding factors.

**Table 4:** Relationships between the number of psychotropic molecules taken per day (0 vs ≥1 or ≤ 1 vs ≥2) and impairment in cognitive and mobility performance (logistic regression analysis).

Model	Variable	Number of psychotropic molecules					
		0 vs ≥1			≤1 vs ≥2		
		OR	95% CI	p	OR	95% CI	p
**3.1**	MMSE	2.76	0.42–18.13	.29	10.33	1.96–54.36	**.006**
**3.2**	MoCA	1.89	0.81–4.41	.14	4.42	1.80–10.90	**.001**
**3.3**	TMT A	1.29	0.49–3.39	.61	2.47	0.94–6.55	.07
**3.4**	TMT B	0.88	0.28–2.81	.83	2.97	1–9.14	**.05**
**3.5**	TMT B-A	0.75	0.18–3.19	.70	5.76	1.67–19.80	**.005**
**3.6**	TUG	1.03	0.43–2.44	.96	3.87	1.52–9.88	**.005**

Different models were used to analyze the links between the number of psychotropic molecules taken and impaired MMSE (Model 3.1), impaired MoCA (Model 3.2), impaired TMT B (Model 3.3), impaired TMT B-A (Model 3.3), and impaired TUG (Model 3.5). All models were adjusted for covariates: age, education level, and comorbidities for MMSE; comorbidities and age only for MoCA (standard scores already adjusted for education level); comorbidities only for TMT scores; and BMI, handgrip strength, comorbidities, and risk of falls for TUG. Impaired scores on TMT B and TMT B-A were already stratified by age and education (Nasreddine et al., 2005), and impaired TUG scores, by age (Sanchez-Cubillo et al., 2009).

Moreover, as summarized in **Table 5**, when the MMSE and TUG scores (Model 4.1) or the MoCA and TUG scores (Model 4.2) were included in the same model, each impaired score was significantly and independently associated with the use of at least two daily psychotropic molecules. The Model 4.3, that included the TUG and TMT B scores, was not significant. Finally, when the TMT B-A and TUG scores were included in the same multivariate model (Model 4.4), only the impaired TMT B-A and one covariate (handgrip) were significantly, and independently, associated with the use of at least two daily psychotropic molecules; no interaction was found between TMT B-A and handgrip. Psychotropic polypharmacy would thus mainly affect TUG performance through the impaired TMT B-A.

**Table 5:** Interactions between cognition and mobility deficits and the number of psychotropic molecules taken per day (0 vs ≥1 or ≤ 1 vs ≥2) (univariate multinomial logistic regression analysis).

Model	Variable*	Number of psychotropic molecules					
		0 vs ≥1			≤ 1 vs ≥ 2		
		OR	95% CI	*p*	OR	95% CI	*p*
**4.1**	MMSE	5.15	0.49–54.05	.17	20.65	2.20–193.83	**.008**
	TUG	0.99	0.40–2.45	.98	2.80	1.03–7.63	**.045**
**4.2**	MoCA	2.16	0.89–5.28	.09	3.42	1.30–9.00	**.013**
	TUG	0.94	0.39–2.27	.89	3.53	1.32–9.46	**.012**
**4.3**	TMT B	0.88	0.25–3.05	.84	2.34	0.69–8.00	.17
	TUG	0.73	0.29–1.85	.50	2.56	0.93–7.04	.07
**4.4**	TMT B-A	0.72	0.15–3.42	.68	4.94	1.21–20.16	**.026**
	TUG	0.75	0.29–1.9	.54	2.05	0.72–5.87	.18
	Handgrip	0.98	0.94–1.04	.55	0.93	0.86–0.99	**.047**

Different models were used to analyze the links between the number of psychotropic molecules taken and impaired MMSE and TUG scores (Model 4.1), impaired MoCA and TUG scores (Model 4.2), impaired TMT B and TUG scores (Model 4.3), and impaired TMT B-A and TUG scores (Model 4.4). All models were adjusted for covariates: age, education level and comorbidities for MMSE; comorbidities and age only for MoCA (standard scores already adjusted by education level); comorbidities only for TMT scores; and BMI, handgrip strength, comorbidities, and risk of falls for TUG. Impaired scores on TMT B and TMT B-A were already stratified by age and education level (Nasreddine et al., 2005), and impaired TUG scores, by age (Sanchez-Cubillo et al., 2009). Only covariates that reached statistical significance are listed in the table.

## Discussion

The present findings highlight the adverse effects of psychotropic drugs, particularly those resulting from concomitant daily use of several psychotropic molecules, on both cognition and mobility. Impaired mobility and global cognition are both significantly correlated with the number of psychotropic drugs taken, regardless of the psychotropic class. Furthermore, logistic regressions showed that the threshold of two psychotropic molecules per day, identified by the ROC curve analysis, increases the risk of impaired executive function (TMT B and TMT B-A), global cognition (MMSE and MoCA), and mobility (TUG) scores, independent of confounding factors. Finally, when the cognitive and mobility scores are included in the same multinomial regression model, taking at least two psychotropic molecules is significantly and independently associated with concomitant impairments in global cognition (MMSE or MoCA) and TUG. Taking at least two psychotropic molecules is also significantly associated with impaired TMT B-A, but not with impaired TUG.

Poorer performance in global cognition and executive functions with increased use of psychotropic molecules adds strength to the adverse effects of these drugs on psychomotor function, concentration, attention, and memory reported in several large-scale population-based studies (Brooks and Hoblyn, 2007). Most of these studies focused on specific populations, in particular on psychiatric disorders, in which cognitive dysfunction is commonly encountered. Because participants of the present study had essentially normal cognition, our results extend the deleterious cognitive effects of psychotropic drugs to an essentially cognitively intact population.

Adverse effects of psychotropic drugs on balance and gait disorders have received little attention. The poorer basic mobility with the increased number of psychotropic molecules in the present study is, however, in line with the dose-response relationship between the number of psychotropic medications taken and balance impairment reported in a relatively young population aged 40 years and over (Bareis et al., 2018).

A major strength of the present study is the identification of a threshold value of two psychotropic molecules for both impaired cognition and mobility, with a 3- to 10-fold increased risk for cognitive impairment, and a 4-fold increased risk for mobility impairment. This threshold of two psychotropic molecules indicates that psychotropic drugs are highly involved in the risk for impaired mobility and global cognition following polypharmacy (Langeard et al., 2016). Furthermore and interestingly, this threshold of two psychotropic molecules was found here in a relatively healthy population that included both young and old seniors, and for all impaired cognitive and mobility scores, independent of confounding factors, including comorbidities. We may consider the question of whether this would also apply to younger adults.

Despite common prescription of multiple psychotropic medications in various populations (e.g., psychiatric, elderly, dementia, and community-dwelling population) (Hartikainen et al., 2005; Brett et al., 2017; Nørgaaard et al., 2017; Rhee and Rosenheck, 2019), very few studies focused on the effects of psychotropic polypharmacy. Nevertheless, an association between the use of multiple psychotropic drugs and falls in older adults has been reported. Compared to non-users, older adults taking two or more psychotropic drugs are almost twice as likely to experience recurrent falls (Hanlon et al., 2009), and users of four psychotropic drugs have an increased risk of fall injuries, hospitalization, and even death in community-dwelling populations (Johnell et al., 2016). Consistent with these data, the Swedish National Board of Health and Welfare discourages prescribing three or more psychotropic drugs in older patients (Johnell et al., 2007). The present study suggests that physicians should even be cautioned when prescribing as few as two psychotropic molecules.

Looking at the relationships between cognition and mobility with psychotropic polypharmacy consumption provides other important findings. Thus, consuming two or more psychotropic molecules impaired global cognition and mobility in an independent manner. Interestingly, a similar finding has been reported for polypharmacy, with a cut-off of 5 medicinal molecules (Langeard et al., 2016). This was, however, not the case when focusing on executive function rather than on global cognition. Indeed, our data suggest that gait disorders observed when consuming two or more psychotropic drugs would be the consequence of an executive dysfunction. This further suggests that psychotropic polypharmacy would preferentially affect executive function, which in turn would induce gait disorders. It is noteworthy that this was found with the TMT B-A score only, that is a more appropriate measure of executive function than completion time on the TMT-B (Sanchez-Cubillo et al., 2009), which did not reach significance.

In light of the close relationship between gait, falls, and cognition (Muir et al., 2012), and the preferential role of poor executive function in falls and gait abnormalities (Herman et al., 2010; Hsu et al., 2012), these findings suggest that impaired executive function following the use of psychotropic polypharmacy could explain some of the falls reported with multiple psychotropic drugs (Ming and Zecevic, 2018). Prospective studies on falls risk with concomitant in-depth assessment of cognitive and mobility performance would be useful to confirm our hypothesis.

It is noteworthy that the cut-off of two psychotropic molecules for the risk of impaired cognition and mobility, as well as the dependency of TUG impairment upon the executive dysfunctioning when consuming two or more psychotropic molecules, were found regardless of the psychotropic class. Subclass analyses could not be performed due to the small sample size per subclass. However, univariate multinomial logistic regression analysis focusing on the four subclasses most consumed by the participants (i.e. antidepressants, analgesics, hypnotics, and anxiolytics that were each taken by at least 17 participants) indicated that no psychotropic subclass appears to be significantly associated with impaired cognition and mobility (data not shown). This would be consistent with the lack of relationship recently reported between individual classes of psychotropic medications and balance impairment (Bareis et al., 2018). More studies are nevertheless required to explore the putative involvement of specific psychotropic classes in people at risk for cognitive or mobility impairments.

Some limitations can be addressed. First, one could argue that the present findings may not be relevant to the general population since all participants had fallen in the previous year. Nevertheless, as previously discussed in detail (Langeard et al., 2016), our population is very similar to the general population: a harmless fall in a large number of participants, and mean cognitive and mobility scores within the normal range. In addition, because 30% of individuals older than 65 years fall every year (Hopewell et al., 2018), and since we specifically searched for individuals who had fallen in the previous year, after our 5 years of inclusion we had likely collected a significant part of the general population. Regarding the small number of men in our sample, it is inherent to the lower number of men than women admitted to the hospital, and who experienced a fall with a low-energy fracture (21% in the initial description of the osteoporosis cohort of the University hospital in Caen (Levasseur et al., 2007) and 16% during the inclusion period of the present study), together with the well-known fact that men are less likely to agree to participate in this type of research (Markanday et al., 2013). Moreover, although our sample size is relatively small, the results of the multivariate logistic regression can be considered as robust because the confidence intervals are relatively narrow except for the MMSE and TMT B-A for which the lower limit confidence interval is, nonetheless, close to two. It would, however, be appropriate to confirm these results in a larger population, including more men if possible, and in a non-falling population. Nonetheless, it should be noted that a strong association has been reported between gait disorders and polypharmacy (George and Verghese, 2017) as well as between cognitive impairment and polypharmacy (Gnjidic et al., 2012) among non-falling community-dwelling adults. Second, we did not separately analyze the effects of the psychotropic molecules according to their specific action on the central nervous system (i.e. depressants, stimulants, or sedatives) due to the small population sample. Despite this fact, the present findings reflect the reality in terms of the use of psychotropic polypharmacy in the general population. Third, there could have been *a protopathic bias since* some psychotropic medications could have been prescribed for cognitive impairment; however, less than 8% of the participants had impaired MMSE, and only 4% of the prescribed psychotropic molecules were anti-dementia molecules. Finally, dose and duration of treatment were not considered, which would, however, be useful in future studies.

## Conclusion

The present study indicates that community-dwelling adults 55 years and older are at risk for both mobility and cognitive impairments when as few as two psychotropic molecules are consumed. Prospective studies would be useful to determine whether this threshold is similar or lower after long-term use of psychotropic molecules. The present findings also suggest that gait disorders observed when consuming two or more psychotropic molecules would mainly be the consequence of an executive dysfunction, which could further lead to falls. Such adverse effects of psychotropic polypharmacy in relatively healthy and young-old adults should alert physicians when prescribing combinations of psychotropic molecules. Health policy makers should also be aware of these findings in order to implement appropriate actions to alert prescribers of psychotropic polypharmacy.

## Data Availability Statement

The datasets generated for this study are available on request to the corresponding author.

## Ethics Statement

The Lower Normandy Ethics Committee approved the present study (no. 2011A00556-35; clinical trial registration number: NCT02292316). The patients/participants provided their written informed consent to participate in this study.

## Author Contributions

Study concept and design, and obtaining funding: CC and CM. Study supervision and integrity of the data: CC. Acquisition of subjects and/or data: GL, PL, KP, and EA-M. Analysis and interpretation of data: CC, GL, M-LB, and EA-M. Preparation/Critical review of the manuscript: All authors. Statistical expertise: RM.

## Funding

This work was supported by the French Ministry of Health (PHRC, Programme Hospitalier de Recherche Clinique 2011 no. 2011-A00534-37). EA-M by the Togolese Ministry of Higher Education and Research (No. 25/MESR/SG/DBS), and the Normandy Association for Physiology Research (ANDREP), and KP by the PHRC, the Regional Council of Lower Normandy, and the GRAAL association (Groupe de Recherche sur les Affections de l’Appareil Locomoteur).

## Conflict of Interest

The authors declare that the research was conducted in the absence of any commercial or financial relationships that could be construed as a potential conflict of interest.
